# Development of Cashew and Pistachio Ladders through a Food-Processing Approach

**DOI:** 10.3390/foods13213440

**Published:** 2024-10-28

**Authors:** Nicki Shwe Yee, Hoi Ka Ng, Jingjing Zeng, Jinjing Bao, Dianne E. Campbell, Paul J. Turner, Nanju Alice Lee

**Affiliations:** 1 School of Chemical Engineering, University of New South Wales, Kensington, NSW 2052, Australia; w.shweyee@unsw.edu.au (N.S.Y.); hoika.ng@unsw.edu.au (H.K.N.); jingjing.zeng1@alumni.unsw.edu.au (J.Z.); jinjing.bao@alumni.unsw.edu.au (J.B.); 2 Sydney Medical School, Faculty of Medicine & Health, University of Sydney, Sydney, NSW 2006, Australia; dianne.campbell@sydney.edu.au; 3 Immunology and Allergy, Children’s Hospital at Westmead, Westmead, NSW 2145, Australia; 4 Centre for Food Allergy Research (CFAR), Murdoch Children’s Research Institute, Melbourne, VIC 3052, Australia; 5 National Heart & Lung Institute, Imperial College London, London SW3 6LY, UK; p.turner@imperial.ac.uk

**Keywords:** cashew, pistachio, cross-reactivity, boiling, soaking, allergenicity, hypoallergenic, IgE

## Abstract

Following successful oral immunotherapy (OIT) for peanut allergy using boiled peanuts (BOPI trial), this study investigated the potential of wet-thermal-processing-induced allergen modification, specifically soaking and boiling (1–4 h) to reduce the allergenicity of cashew and pistachio allergens. In addition, this study provides a foundation of understanding for developing safer forms of cashew/pistachio administration for application in OIT by gradual exposure to increasing doses of modified allergens with reduced potency as an “allergen ladder”. An SDS-PAGE analysis and an intrinsic-fluorescence spectroscopy revealed altered tertiary structures of the allergens, leading to their denaturation and even degradation. Notably, the reduction in both allergen-specific polyclonal IgG and human-specific IgE (sIgE) binding correlated with the treatment time, with the most significant decrease observed after 4 h of boiling. In contrast, shorter soaking treatments showed negligible effects on the IgE-binding capacity of these nuts, therefore indicating a further need for optimization. These findings indicate that extended boiling effectively reduced the amounts of the highly potent allergenic component Ana o 3 in cashew and Pis v 1 in pistachio, as confirmed by ELISA using polyclonal anti-Ana o 3 antibodies, and an immunoblot showed decreased IgE epitope binding in cashew and pistachio allergens, which further modified their allergenic profiles. This approach shows promise as a viable method for offering a safer therapeutic option for cashew/pistachio allergy.

## 1. Introduction

Food allergies have been reported worldwide, and tree nuts are one of the major food groups responsible for such adverse reactions. Tree nuts are a potent source of allergenic proteins that trigger IgE-mediated hypersensitivity, leading to severe and potentially life-threatening anaphylactic reactions [1,2]. Despite these considerations, the consumption of tree nuts is on the rise, particularly in industrialized countries, due to their well-recognized health benefits [1,2]. The prevalence of tree nut allergy varies with age, definition of food allergy, and geographical area, among other factors, ranging from less than 1% to 3% globally [3].

Cashew and pistachio nuts are among the tree nuts causing IgE-mediated allergies. Both cashew nuts and pistachios belong to the same botanical family (*Anacardiaceae*); they are phylogenetically related and have been known to cause co-sensitization and cross-reactivity [4]. The prevalence of cashew nut allergies seems to be rising, which correlates with its increasing consumption. A systematic review conducted by McWilliam et al. [3] showed the prevalence of cashew, the most common tree nut allergy, was reported in the USA. There are limited number of studies reporting the prevalence of tree nuts in Europe. A recent systematic review reported the prevalence of tree nut allergy/sensitization to individual tree nuts where overall point prevalence for SPT positive cashew nut allergy was 0.8% [5]. However, no information on cashew/pistachio allergy could be retrieved from emerging economies like Asia and Africa.

Currently, there is no cure for tree nut allergies. The most effective management of food allergies remains strict avoidance. Additionally, omalizumab, an anti-IgE antibody, has been approved as both a standalone treatment and as an adjunct to OIT for food allergy in the US, receiving approval by the Food and Drug Administration in early 2024 [6]. Prior to this, omalizumab had already been widely recognized and approved for treating other allergic disorders, including asthma and chronic urticaria, in several countries such as US, EU, Australia, Canada, and Colombia. Thoroughly avoiding exposure to the offending agent is, however, challenging due to the high prevalence of nuts as ingredients in processed food products and possible mislabeling and cross-contact during food processing. Recently, oral immunotherapy (OIT) has emerged as a promising treatment option for food-allergic patients. However, frequent allergic reactions (mostly mild, but occasionally anaphylaxis) are significant side effects associated with using allergenic food sources in the therapy. This makes participants adhering to the therapy regime difficult, and about 20% are unable to continue with their immunotherapy [7]. To reduce severe allergic reactions, modified allergens have been applied to oral immunotherapy as a safer therapeutic reagent [6,7,8,9,10]. For example, boiled peanuts were introduced in the Boiled Oral Peanut Immunotherapy (BOPI) study (NCT02149719) that successfully induced desensitization, with >50% demonstrating sustained unresponsiveness after stopping peanut consumption for 4 weeks [10]. A similar approach involved stepwise reintroduction of food allergens, referred to as a “ladder”, starting with extensively heated forms, which have reduced allergenicity, and gradually progressing to less heated forms, while also increasing the protein amount at each stage. Which allows for controlled desensitization and reduces the risk of allergic reactions [11].

A comprehensive understanding of the impact of food-processing methods on food allergen structures and serum IgE epitopes is critical for developing suitable hypoallergenic products for immunotherapy with safer profiles. The effect of food processing on protein functionality and allergenicity is complex and depends on a variety of factors, such as types of proteins, types of processing methods, and specific conditions employed.

Various food-processing techniques have been reported to influence allergenic properties of tree nut allergens. Dry and wet thermal treatments, such as roasting, boiling, and autoclaving, have been shown to reduce the sIgE-binding capacity [9,12]. Novel non-thermal processing treatments, such as cold plasma, ultrasound, Gamma radiation, and high-pressure processing [8,13], have also been observed to progressively diminish the allergenic potential of tree nut allergens [8,9]. Among the studied food-processing techniques, enzymatic fermentation and chemical hydrolysis show significant promise in reducing the allergenic profile of milk and soy to the extent that they may not elicit symptoms [14]. Furthermore, combining thermal treatments with other chemical and biological processing techniques could offer an advanced strategy for producing hypoallergenic foods [10,15]. Indeed, Cuadrado et al. demonstrated that pressured-heating techniques, such as autoclave and controlled instantaneous depressurization combined with enzymatical treatment, are highly effective to obtain hypoallergenic peanuts and tree nuts such as pistachio, cashew, and hazelnut [16,17,18]. While cashews and pistachios boiled for up to 1 h have shown a reduction in IgE reactivity through chemical and functional modifications [3], the effects of extensive boiling have not been thoroughly studied. Soaking, a common tree-nut-processing technique used to initiate enzyme activation [3], combined with boiling could facilitate more desirable modifications of allergenic proteins.

Building on the success of peanut OIT, BOPI, this study aimed to investigate the effects of soaking and boiling, both individually and in combination, on the protein structure, solubility, and antigenic and allergenic integrity of cashew and pistachio allergens. Additionally, it aims to determine the optimal conditions for producing cashew and pistachio ladders suitable for OIT [11]. The findings of this study contribute to the knowledge base of food allergology and provide valuable insights into potential food-processing methods to develop safer therapeutic agents for cashew and pistachio allergy immunotherapy.

## 2. Materials and Methods

### 2.1. Chemicals

Hexane, sodium chloride (NaCl), along with other chemicals for electrophoresis dithiothreitol (DTT), 10× Tris/Glycine/SDS buffer, Coomassie brilliant blue-R 250, and methanol (HPLC grade) were purchased from Sigma-Aldrich (St. Louise, MO, USA). Precision Plus Protein Dual Colour Standard and Trans-Blot^®^ Turbo ™ 5× transfer buffer were obtained from Bio-Rad (Hercules, CA, USA). Bovine serum albumin-BSA protein standard (2 mg/mL), bicinchoninic acid (BCA) solution, copper (II) sulphate solution, gelatin from cold water fish skin, tetramethylbenzidine (TMB), Tris-HCl-0.9% NaCl buffer (TBS), TWEEN^®^ 20, and Anti-sheep IgG A3415 were obtained from Sigma-Aldrich. Goat anti-rabbit IgG (HRP) ab6721 was obtained from Abcam (Cambridge, UK). A SuperSignal^®^ West Dura extended-duration substrate was obtained from Thermo Fisher Scientific (Waltham, MA, USA). Mouse anti-human IgE Fc-HRP 9160-05 was obtained from Southern Biotech (Birmingham, AL, USA).

### 2.2. Human Serum Samples and Cashew- and Ana o 3-Specific Polyclonal Antibodies

Ten patient serum samples, obtained from two sources, were pooled for the IgE-binding study. Eight samples were selected from the baseline samples of the “Boiled Peanut Oral Immunotherapy” (BOPI) pilot study (Trial number NCT02149719, human ethics approval 15/LO/0287) at Imperial College London, United Kingdom (UK). These samples were from children aged 8–14 years who were diagnosed with peanut allergy via a double-blind, placebo-controlled food challenge (DBPCFC). They were also clinically confirmed to be cashew- and pistachio-sensitized via skin prick test (wheal diameter for cashew 5–15 mm; and for pistachio 4–12 mm). Of the eight samples, two had previous reactions to either cashew or pistachio.

Two additional samples were obtained from the Children’s Hospital at Westmead, Australia, where the patients were clinically diagnosed either as cashew/pistachio-sensitized or allergic based on a convincing history of ana-phylaxis (systemic urticaria and respiratory symptoms upon consumption) and positive SPT results (cashew 11–13 mm; pistachio 3.5–9 mm) (human ethics approval #LNR/14/SCHN/516). Detailed information on the patient serum samples is in Supplementary Table S1.

The rabbit anti-Ana o 3 antibody was produced at the IMVS Veterinary Services Division (South Australia) and sheep anti-cashew protein antibody was produced at the Antibodies Australia in Monash University (Victoria) using as a standard immunization protocol with modification. The purification of Ana o 3, immunization schedules, and serum collection are detailed in [19].

### 2.3. Soaking and Boiling Treatments of Cashew and Pistachio Nuts

Raw and roasted whole cashew and pistachio nut kernels were purchased from a local supermarket in Sydney, NSW, Australia. The raw and roasted whole kernels were washed with reverse osmosis (RO) water, which involves pressure-forcing water through a semi-permeable membrane to remove contaminants (e.g., salt), before applying different process treatments (i.e., soaking and boiling). For the soaking study, the nuts were immersed in RO water (1:5 *w*/*v*) for 30 min (at 37 °C and RT), 1 h, and 2 h at room temperature (RT). The aliquots of water (referred to as soaked water hereafter) were collected, and the nuts were air-dried. For the boiling study, the nuts were boiled in RO water (1:5 *w*/*v*) at 99–100 °C for 30 min, 1 h, 2 h, and 4 h. The aliquots of water (referred to as boiled water hereafter) were collected, and the boiled kernels were freeze-dried. The protein in the soaked-water samples was concentrated by centrifuging in the 3K Amicon^®^ Ultra Centrifugal Filter Units (Merck, Burlington, MA, USA) at 5000× *g* for 30 min.

### 2.4. Protein Extraction

Cashew extract was prepared according to the method of Zhao et al. [19]. The untreated and soaked kernels were ground using a commercial coffee grinder and defatted with *n*-hexane (1:5 *w*/*v*). All defatted flours were air-dried overnight to remove residual solvent. Soluble proteins from the defatted flour were extracted in a Tris-HCl buffer solution (0.1 M Tris-HCl with 0.9% NaCl, pH 8.4) in a ratio of 1:10 *w*/*v* with constant agitation overnight at 4 °C. Following the overnight extraction, the mixture was centrifuged at 10,000× *g* for 10 min at 4 °C, and the supernatants were collected as soluble protein extracts. The protein concentrations of the extracts, soaking and boiling water, and the concentrated soaking water were determined using bicinchoninic acid assay (BCA), t. Unprocessed cashew and pistachio nuts were set as control (raw nuts).

### 2.5. Sodium Dodecyl Sulphate-Polyacrylamide Gel Electrophoresis (SDS-PAGE)

The electrophoretic profile of the cashew and pistachio protein extracts (both heat-treated and -untreated), soaked-water sample, and boiled-water samples were determined with SDS-PAGE, using either the same volume or the same protein quantity for different purposes. For the cashew samples, when loading the same volume of samples onto the gel, untreated soaked-water samples were diluted 1:5 with phosphate-buffered saline (PBS, pH 7.4), while boiled-cashew proteins were diluted 1:2 with the same buffer. For the pistachio samples, both treated and untreated pistachio proteins were diluted to 1:5 with Tris-HCl buffer solution (0.1 M Tris-HCl with 0.9% NaCl, pH 8.4) before loading onto the gel. For the SDS-PAGE, where the same quantity of protein was loaded onto the gel, all samples (i.e., untreated soaked and boiled, as well as treated water) were diluted to 1 mg/mL, where 10 μL mixed the protein extracts samples, and 15 μL mixed the treated-water samples for the same protein amount with SDS-PAGE gel for 7 μg of proteins of each sample.

For both SDS-PAGEs, the diluted samples were mixed with a sample buffer (3:100 *w*/*v* ratio of DTT) and 2× Lammeli buffer, Bio-Rad, USA) in a 1:1 *v*/*v*, heated at 95 °C for 5 min, centrifuged at 10,000× *g* for 5 min, and electrophoresed in 15% hand-casted gels. After electrophoresis, gels were fixed in fixing solution (10% methanol and 7% acetic acid) for 30 min and then stained overnight in staining solution (4% methanol and 1% acetic acid with Coomassie brilliant blue) with constant agitation. Following the overnight staining, the gels were destained in a destaining solution (40% methanol and 10% acetic acid) for 2 to 3 h, with constant shaking, before visualizing the electrophoretic protein patterns by using a ChemiDoc XRS+ system Bio-Rad (Hercules, CA, USA).

### 2.6. Intrinsic-Fluorescence Spectroscopy

The intrinsic-fluorescence spectra of the cashew and pistachio protein extracts, as well as the treated-water samples, were characterized with a Cary Eclipse fluorescence spectrophotometer Agilent Technologies (Santa Clara, CA, USA). Briefly, 0.5 mg/mL of the protein extracts of the treated and untreated cashew and pistachio nuts and treated soaked and boiled water were scanned under an excitation fluorescence wavelength of 280 nm and the emission wavelength at 300 to 450 nm, with both excitation and emission slits of 10 nm.

### 2.7. Inhibition Immunoassay Using Allergen-Specific Polyclonal IgG

For the inhibition immunoassay using allergen-specific polyclonal IgG, animal sera from sheep and rabbits immunized with crude protein and Ana o 3, respectively, were used for both cashew and pistachio samples. Briefly, Nunc MaxiSorp™ 96-well plates Thermo Fisher (Waltham, MA, USA) were coated with treated and untreated cashew and pistachio protein extracts that were diluted to a final concentration of 0.1 μg/mL with coating buffer (50 mM NaHCO_3_ buffer, pH 9.6) by incubating overnight. The wells were washed with 0.05% Tween 20 and blocked with blocking buffer (0.5% fish gelatin in PBS, pH 7.8) for 1 h.

In separate 1.2 mL cluster tubes (Corning Inc., Corning, NY, USA), animal sera containing polyclonal IgG were pre-incubated with untreated and treated allergen extracts (from 0.01 ppm to 100 µg/mL) for 30 min. For blank and negative control, blocking buffer and animal sera pre-incubated with the blocking buffer were used, respectively. In total, 150 μL of the pre-incubated sample solutions, blank sample, and negative control sample were transferred to the coated microwell plate and incubated for 1 h. The wells were washed five times with 0.05% Tween 20, and 100 μL of diluted conjugated secondary antibodies (donkey anti-sheep IgG and goat anti-rabbit IgG) were incubated for 1 h. For the development of colour, 100 μL of substrate/chromogen solution (TMB and H_2_O_2_) per well was incubated for 30 min. The reaction was then stopped by adding 50 μL 0.125 M sulfuric acid. The absorbance values of microwells were measured at 450 nm using a SpectraMax M2 microplate reader (Molecular Devices, San Jose, CA, USA). The percent inhibition % was calculated with the following formula:

$$Inhibition\;\%=\left[1-\left(\frac{{Ab}_{sample}}{{Ab}_{negative}}\right)\right]\times 100$$

where *Ab_sample_* is the average absorbance value of samples (i.e., allergen extracts), while *Ab_negative_* is the averaged absorbance of negative control. All *Ab_sample_* and *Ab_negative_* values were adjusted with the averaged blank absorbance value.

### 2.8. sIgE-Based Immunoblot Analysis

For sIgE dot-blot analysis, all allergen extracts (untreated and treated) and treated-water samples were diluted with TBS to a final protein concentration of 1 mg/mL. Each sample (1 μL) was dotted and air-dried onto a nitrocellulose membrane. The membrane was blocked with a blocking buffer (3% BSA-TBS, pH 7.5) overnight, with constant agitation. The membrane was then washed three times with 0.05% Tween 20 and incubated with diluted human serum (1:50 *v*/*v*) overnight. The membrane was washed 5 times with 0.05% Tween 20 and a final rise with MilliQ water. It was incubated with mouse anti-human IgE-HRP (Southern Biotech, Birmingham, AL, USA) for 1 h. Finally, the membrane was washed three times with 0.05% Tween 20. A chemiluminescence substrate (SuperSignal^®^ West Dura Extended Duration Substrate, Thermo Fisher, USA) was added, and the IgE-binding was visualized using a ChemiDoc MP System (Bio-Rad^®^, USA).

For IgE immunoblot, the protein extracts of untreated- and treated-cashew and -pistachio samples, as well as the treated-water samples, were electrophoresed using 15% hand-casted SDS-PAGE gels. The proteins were separated for 1 h at 150 V using a Mini-PROTEAN electrophoresis system (Bio-Rad, Hercules, CA, USA). The separated proteins were transferred onto a nitrocellulose membrane with a Trans-Blot Turbo Transfer System (Bio-Rad, Hercules, CA, USA) at 25 V for 7 min. After transferring, the membrane was washed 3 times with 0.05% Tween 20, followed by final rising with Milli Q water. The membrane was blocked with a 1% BSA-TBS (pH 7.5) overnight, with constant agitation. The remaining steps after the blocking step followed the same steps of IgE dot-blot analysis as described above.

### 2.9. Statistical Analysis

All experiments were conducted in triplicates. Statistical analyses were performed using GraphPad Prism 9.3.1, and one-way ANOVA was used to determine statistical significance (*p* < 0.05). The numerical data are presented as mean values with standard error of the mean (SEM).

## 3. Results and Discussion

### 3.1. Effects of Food-Processing Treatments on Solubility and Molecular Profiles of Treated-Nut Proteins

Tree nuts are commonly associated with food-allergy-related adverse reactions and anaphylaxis. Accordingly, this study investigated the effects of thermal treatments such as soaking and extensive boiling on the protein structure integrity, solubility, and allergenic properties of cashew and pistachio allergens. By subjecting these tree nut proteins to controlled thermal processes, we can understand the essentials for developing safer allergy management strategies and could contribute to reducing the health risks associated with nut consumption.

#### 3.1.1. Effects of Soaking on the Solubility and Profile of Proteins in Cashew and Pistachio Kernels

**Figure 1:**
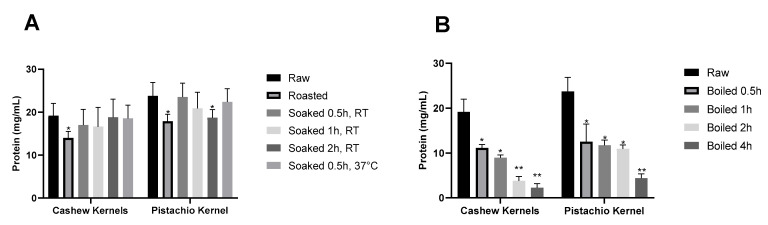
Concentrations of proteins in (**A**) raw, commercially roasted, soaking-treated, and (**B**) boiled cashew and pistachio kernels. *p* values: (** <0.001, * <0.01) are indicated.

**Figure 2:**
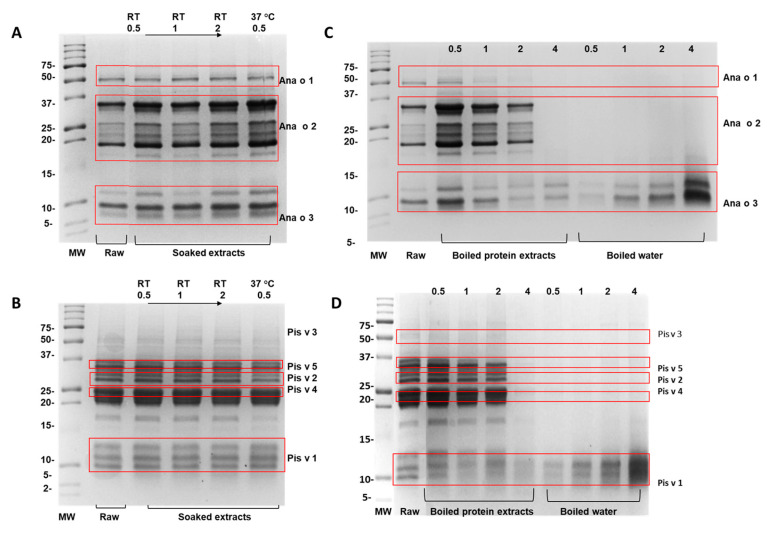
The SDS-PAGE showing the protein profiles of soaked (**A**) cashew and (**B**) pistachio, boiled (**C**) cashew and (**D**) pistachio kernels, water samples, and raw kernel extracts.

Processing allergenic ingredients through wet thermal processing such as soaking is typically intended to soften the seed by increasing its water uptake and removing low-molecular-weight allergens [20]. Accordingly, the effects of soaking at room temperature versus an elevated temperature, 37 °C, on the solubility and extractability of cashew and pistachio proteins were studied. The protein concentrations of the extracts of raw, soaked, and roasted cashew and pistachio kernels are presented in Figure 1A. The protein profiles of soaking-treated cashew and pistachio kernel proteins are presented in Figure 2A and Figure 2B, respectively. As apparent from Figure 1A, the soaking temperature and time had no significant effects on the extractable protein concentrations in both cashew and pistachio kernels. Overall, raw nuts exhibited the highest soluble protein concentrations (19.2 mg/mL and 23.8 mg/mL for cashew and pistachio, respectively) in contrast to the roasted nuts, which exhibited the lowest soluble protein concentrations in both cashew and pistachio (14 mg/mL and 18 mg/mL, respectively). Soaking the cashew kernels resulted in a slight decrease in soluble protein concentrations (i.e., 17 mg/mL when soaked for 0.5 h at RT and 16.6 mg/mL when soaked for 1 h at RT). Interestingly, neither soaking time nor temperature significantly impacted the soluble protein concentrations in cashew kernels (i.e., 18.8 mg/mL when soaked for 2 h at RT and 18.6 mg/mL when soaked for 0.5 h at 37 °C). On the other hand, pistachio kernels showed markedly decreased extractable protein concentrations with soaking time (i.e., 23.5 mg/mL when soaked for 0.5 h at RT versus 18.7 mg/mL when soaked for 2 h at RT). However, soaking temperature did not impact the soluble protein concentrations (i.e., 22.4 mg/mL when soaked for 0.5 h at 37 °C). Therefore, it was concluded that overall, soaking did not significantly alter the protein concentration of both cashew and pistachio nuts in the current study.

The electrophoretic profiles of cashew and pistachio further complemented the findings that the soaking treatment did not significantly impact the protein profiles of these nuts (Figure 2A,B). Overall, the similar intensity of each protein band illustrated that the protein concentrations for cashew nut kernels soaked under different conditions (i.e., 0.5 h, 1 h, 2 h at RT and 0.5 h, 37 °C) were similar (Figure 2A). However, Ana o 2 and Ana o 3 bands appeared to have lower protein content in raw- and soaked-cashew extracts for 1 h at RT. Similarly, the protein profiles and concentrations of pistachio extracts were generally comparable (Figure 2B), and only one protein band of pistachio at around 17 kDa started to fade at 0.5 h soaking at 37 °C. Therefore, it was concluded that soaking did not exhibit obvious effects on protein degradation in the current study.

#### 3.1.2. Effects of Boiling on the Solubility and Profile of Proteins in Cashew and Pistachio Kernels

The extended boiling (1–4 h) induced remarkable reductions in the extractable protein concentrations for both cashew and pistachio in the current study (Figure 1B). In particular, the prolonged boiling at high temperatures reduced the solubility of cashew and pistachio proteins. For both cashews and pistachios, the soluble protein concentration decreased from 19.2 mg/mL and 23.8 mg/mL (raw) to 2.3 mg/mL and 4.4 mg/mL, respectively, when extensively boiled for 4 h (Figure 1B). According to Cuadrado et al., higher protein degradation is achieved when enzymatic hydrolysis after pressured heating is applied in both nuts [16,18]. Compared to the cashew nuts, which exhibited a significant decrease in soluble protein concentrations proportional to boiling time (i.e., from 19.2 mg/mL (raw) to 11.2 mg/mL, 9 mg/mL and 4 mg/mL when boiled for 0.5 h, 1 h, and 2 h, respectively), the pistachio exhibited a more stable protein concentration between boiling times up to 2 h (12.5 mg/mL for 0.5 h and 11 mg/mL for 2 h). Beyond 2 h, there was a significant reduction in soluble proteins (4.4 mg/mL), suggesting pistachio proteins were more resistant to heat.

As with the soaking samples, the protein profiles of the boiled-water samples of cashew and pistachio and raw kernel extracts were characterized using SDS-PAGE (Figure 2C,D). As demonstrated in Figure 2C, the disappearance of Ana o 1 and Ana o 2 bands illustrated that the extensive boiling of cashew kernels for 4 h significantly reduced the extractable protein probably due to decreased protein solubility of 7S globulin and 11S globulin. The concentration of Ana o 3 in boiled kernels was proportionally decreased with boiling time (Figure 2C), which was likely due to structural modifications such as denaturation, aggregation, or reactions with food components. In line with this, the boiled water contained increasing Ana o 3 concentrations from leaching of this allergen into the boiling water, reaching its highest quantity at 4 h. This was in line with previous studies that showed soluble protein leakage from nut kernels into the treated water when peanuts were boiled [21,22].

Furthermore, the most distinct protein bands, approximately 33 kDa (Pis v 5) and 13 kDa (Pis v 1), were observed when the pistachio kernels were boiled for 0.5 h (Figure 2D), similar to previously reported studies [23,24]. Most of the pistachio proteins were resistant to the boiling treatment up to 2 h, indicated by a protein profile similar to that of raw pistachio. Of the two Pis v 5 bands, the higher molecular band was susceptible to boiling from 0.5 h and completely disappeared by 2 h. The lower molecular band of Pis v 5, Pis v 2, and Pis v 4 were generally resistant to boiling for up to 2 h. The boiling treatment also caused protein fragmentation in both cashew and pistachio samples. Similarly to cashew Ana o 3, Pis v 1 also showed leaching effects proportional to boiling time. Boiling for 4 h induced degradation of cashew and pistachio proteins, with high-molecular-weight proteins degrading into low-molecular-weight forms. This finding is consistent with the observation by Sanchiz et al. [25] of protein degradation in both nuts after 30 min of autoclaving at 121 °C.

### 3.2. Intrinsic Fluorescence Spectroscopy of Cashew and Pistachio Nut Proteins

**Figure 3:**
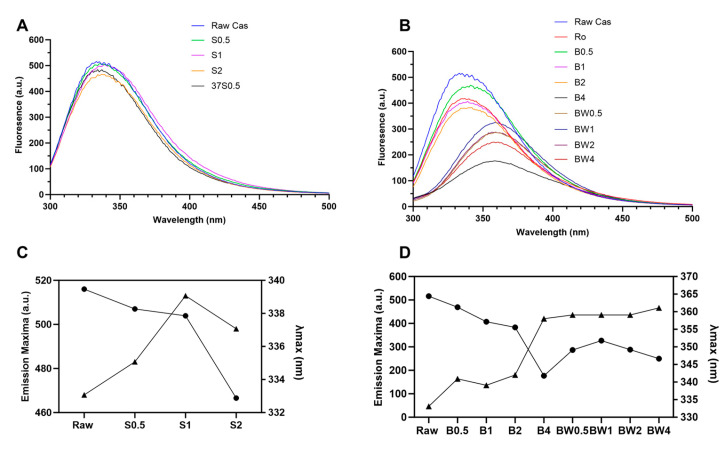
Fluorescence emission spectrum of (**A**) soaking-treated and (**B**) boiled-cashew samples. The maximum fluorescence intensity of cashew protein. (**C**) Fluorescence emission maxima (-•-) and λmax (-▴-) of soaked and (**D**) boiled for 0.5, 1, 2, and 4 h. Cas: cashew; Ro: roasted cashew; S: soaked raw kernels (0.5, 1, and 2 h at RT and 0.5 h soaked at 37 °C); SW: soaked water (0.5, 1 and 2 h); B: boiled cashew (0.5, 1, 2 and 4 h); BW: boiled water (1, 2 and 4 h).

**Figure 4:**
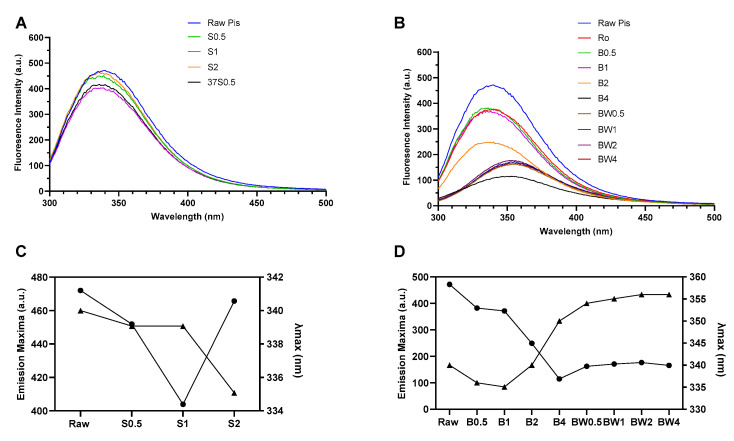
Fluorescence emission spectrum of (**A**) soaking-treated and (**B**) boiled pistachio samples. The maximum fluorescence intensity of pistachio protein: (**C**) fluorescence emission maxima (-•-) and λmax (-▴-) of soaked and (**D**) boiled for 0.5, 1, 2, and 4 h. Pis: pistachio; Ro: roasted; S: soaked; SW: soaked water; B: boiled; BW: boiled water.

The intrinsic fluorescence spectroscopy analysis was used to study the structural changes in soluble proteins in the treated nut kernels and the collected water samples (soaking and boiled water). The fluorescence spectrum of raw kernels with soaking (Figure 3A and Figure 4A) and boiling (Figure 3B and Figure 4B) samples and roasted kernels were analyzed. The relationship between the intensity of emission fluorescence and the duration of soaking (Figure 3C and Figure 4C) and boiling (Figure 3D and Figure 4D) was further illustrated.

Overall, the soaking had minimal effects on the protein structures of both cashew and pistachio kernels (Figure 3B and Figure 4B). Although, the intensity of emission fluorescence started to decrease at 2 h of soaking (Figure 3C and Figure 4C). Therefore, it was concluded that the soaking did not alter the conformational structures of cashew and pistachio proteins.

On the other hand, boiling induced more significant changes in the protein structures of both cashew and pistachio (Figure 3B,D and Figure 4B,D). The boiled cashew and pistachio kernel extracts exhibited red shifts in wavelengths (358 nm and 352 nm, respectively) (Figure 3B and Figure 4B). The fluorescence emission intensities of the boiled cashew and pistachio kernel extracts also exhibited a remarkable decrease as the boiling time increased (Figure 3D and Figure 4D). This observation is in line with previous results, where heat-denatured anacardein was found to expose the previously buried hydrophobic inner core [26]. In particular, 4 h of boiling induced a significant decrease in the fluorescence emission intensities (177 a.u. and 115 a.u., respectively, for cashew and pistachio).

When the protein structure begins to unfold, tryptophan that is originally in the core of the protein gradually becomes exposed to the external water solution, which increases the polarity of the surroundings, leading to a reduced intensity and an increase in the wavelength of protein maximum emission fluorescence [27]. These observations suggested that the native proteins of cashew and pistachio were partially unfolded when boiled for 0.5 h, whereas extensive boiling for 4 h resulted in the fully unfolded state of the cashew and pistachio proteins (Figure 3D and Figure 4D).

### 3.3. Effects of Boiling Treatment on the Antibody-Binding Capacity and Immunoreactivity of Treated Nuts

#### 3.3.1. Inhibition Enzyme-Linked Immunosorbent Assay (ELISA) Using Polyclonal Cashew Specific IgG

To investigate the effects of boiling on the allergenic potential of cashew and pistachio allergens, the current study further investigated the binding of sheep anti-cashew proteins antibodies (IgG) to the raw and treated nuts using an inhibition ELISA. The IC_50_ values were determined as an indicator to represent “a half maximal inhibitory concentration”. The higher IC_50_ values indicated the lower inhibitory ability of the inhibitor to prevent the binding between (untreated) raw proteins and IgG.

**Figure 5:**
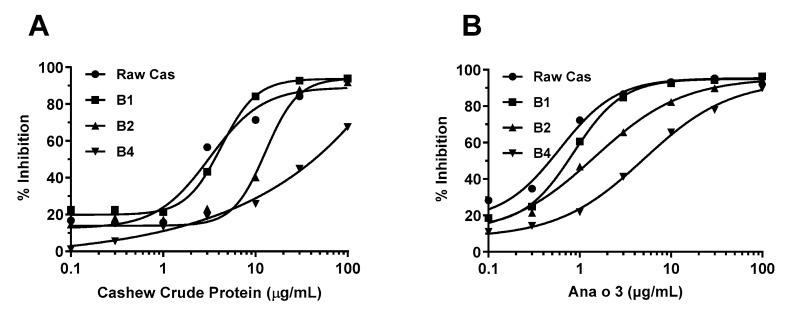
Inhibition of raw cashew protein by heat-treated cashew proteins in the inhibition ELISAs using sheep anti-cashew proteins polyclonal antibodies and rabbit anti-Ana o 3 polyclonal antibodies. (**A**) Inhibition ELISA using sheep anti-cashew proteins antibodies with heat-treated cashew proteins for 1, 2, and 4 h (B1, B2, and B4). (**B**) Inhibition ELISA using rabbit anti-Ana o 3 antibodies with heat-treated cashew proteins for 1, 2, and 4 h. Raw cas indicates raw (untreated) cashew proteins.

As apparent in Figure 5A, the increased boiling time induced a significant decrease in the IgG-binding capacity of cashew allergens. The crude cashew proteins were able to withstand up to 1 h of boiling, and thereafter, the IgG inhibition decreased as the boiling time increased. Consequently, the immunoreactivity of cashew proteins at 1 h, 2 h, and 4 h pf boiling was reduced by 1.4-, 4.1-, and 14-fold, respectively (Figure 5A). Compared to the immunoreactivity of crude cashew proteins, the decrease in the IgG-reactivity to Ana o 3 was less prominent, around half of those for crude proteins, showing 1.4-, 2.4-, and 8.4-fold for 1 h, 2 h, and 4 h boiling, respectively. Despite significant leaking of Ana o 1, as observed in Figure 2C, there remained a significant IgG-reactivity, but it was reduced by 8-fold with 4 h of boiling. Based on these results, it was concluded that boiling cashew nuts for 4 h resulted in the most significant reduction in the IgG-binding capacity, which was in line with the structural modification as characterized above.

**Figure 6:**
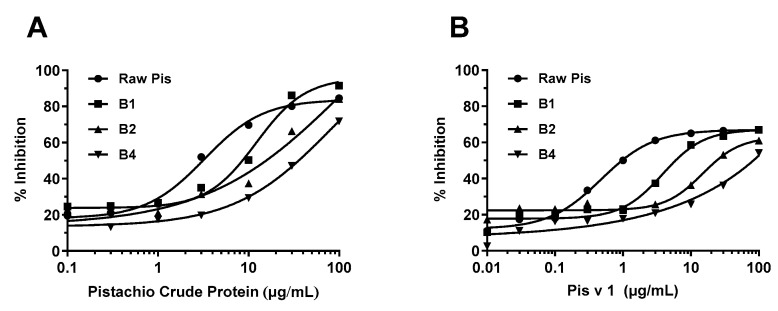
Inhibition of raw cashew protein by heat-treated pistachio proteins in the inhibition ELISAs using sheep anti-cashew proteins polyclonal antibodies and rabbit anti-Ana o 3 polyclonal antibodies. (**A**) Inhibition ELISA using sheep anti-cashew proteins antibodies with heat-treated pistachio proteins for 1, 2, and 4 h (B1, B2, and B4). (**B**) Inhibition ELISA using rabbit anti-Ana o 3 antibodies with heat-treated pistachio proteins for 1, 2, and 4 h. Raw pis indicates raw (untreated) pistachio proteins.

As illustrated in Figure 3D and Figure 4D, the pistachio proteins are more heat-sensitive compared to the cashew proteins, with the peak intensity reduced by 223 a.u. after boiling for 2 h (ΔMax intensity for cashew = 133 a.u.). This was further supported by the significant reduction in the IgG inhibition of the boiled pistachio proteins (Figure 6). In this study, the same polyclonal antibodies specific to cashew proteins/allergens were used, as they were known to have significant clinical cross-reactivity due to the same plant family [25] (Figure 6A,B). Accordingly, the boiled pistachio proteins showed an increasing reduction in IgG inhibition with boiling time, determined by the IC_50_ values, showing a 2.8-fold, 6.1-fold, and 10.7-fold reduction for 1, 2, and 4 h of boiling, respectively. More prominently, the reduction in the inhibition of 2S albumin-specific polyclonal IgG with the boiled pistachio 2S albumin, Pis v 1, was at least 5-fold higher than cashew Ana o 3 (8.1-fold, 31.1-fold, and 208-fold increased on IC_50_ values, respectively). The protein extracts from boiled pistachio demonstrated a consistent reduction in antibody-binding capacity, as apparent from the IgG inhibition ELISA experiments using two polyclonal antibodies specific to crude cashew proteins and Ana o 3, further indicating the cross-reactivity between the allergens of pistachio and cashew.

In the current study, the observed reduction in polyclonal IgG inhibition by both boiled-cashew and -pistachio proteins was consistent with the intrinsic-fluorescence spectroscopy results, which showed that the boiling caused denaturation (as demonstrated by the tertiary structure modifications) and possible loss of conformational epitopes of both cashew and pistachio allergens. Our study hence confirmed that extensive thermal processing (1–4 h of boiling) can significantly reduce the presence of 2S albumin from the cashew and pistachio kernels through leaching. The modification of allergen conformational structures through unfolding/denaturation, or even degradation, led to a subsequent reduction in antibody-binding capacity [13,15].

#### 3.3.2. IgE-Binding of Cashew and Pistachio Proteins

**Figure 7:**
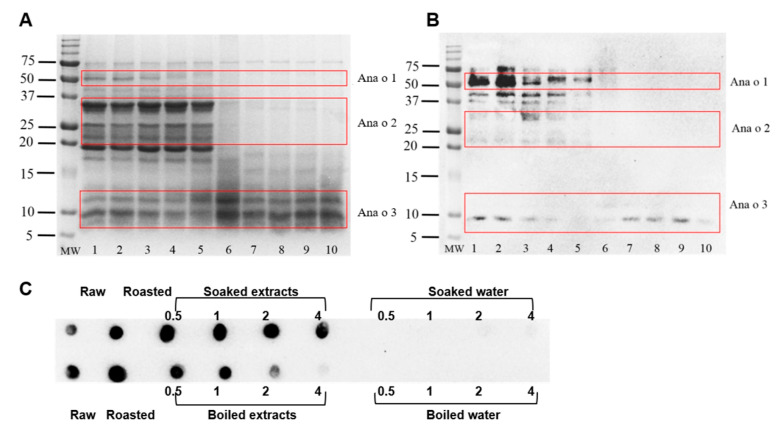
(**A**) SDS-PAGE and (**B**) IgE-immunoblot of cashew protein extracted from raw, roasted, boiled nut and boiled water. MW = standard protein marker. Lane 1 = raw cashew protein extracts. Lane 2 = roasted-cashew-extracted proteins. Lane 3 = 0.5 h boiling kernel proteins. Lane 4 = 1 h boiling kernel proteins. Lane 5 = 2 h boiling kernel proteins. Lane 6 = 4 h boiling kernel proteins. Lane 7 = 0.5 h boiling water. Lane 8 = 1 h boiling water. Lane 9 = 2 h boiling water. Lane 10 = 4 h boiling water. Same loading protein amount for SDS-PAGE. IgE-immunoblot was performed using pooled serum from cashew allergic patients. (**C**) IgE-dot blot of cashew protein extracted from raw, roasted, treated nut, and treated water (indicated in the legend).

**Figure 8:**
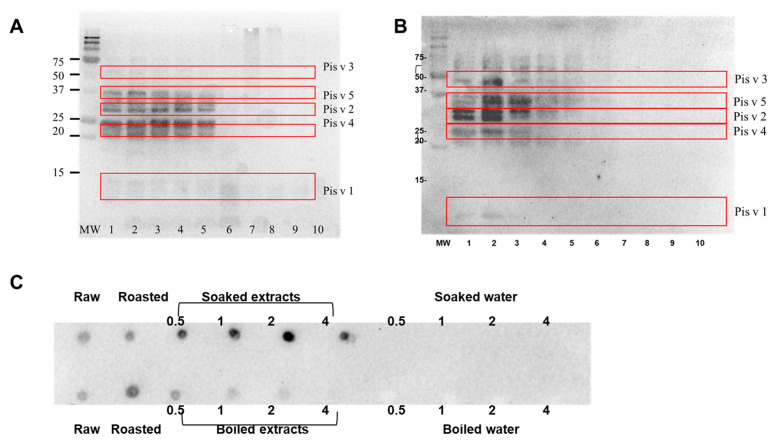
(**A**) SDS-PAGE and (**B**) IgE-immunoblot of pistachio protein extracted from raw, roasted, boiled nut, and boiled water: MW = standard protein marker. Lane 1 = raw pistachio protein extracts. Lane 2 = roasted-pistachio-extracted proteins. Lane 3 = 0.5 h boiling kernel proteins. Lane 4 = 1 h boiling kernel proteins. Lane 5 = 2 h boiling kernel proteins. Lane 6 = 4 h boiling kernel proteins. Lane 7 = 0.5 h boiling water. Lane 8 = 1 h boiling water. Lane 9 = 2 h boiling water. Lane 10 = 4 h boiling water. Same loading protein amount for SDS-PAGE. IgE-immunoblot was performed using pooled serum from cashew allergic patients. (**C**) IgE-dot blot of pistachio protein extracted from raw, roasted, treated nut, and treated water (indicated in the legend).

To further investigate the impact of processing-induced structural modification on the serum sIgE binding of cashew and pistachio allergens, the current study analyzed the IgE-binding capacity using IgE immunoblots and dot blots (Figure 7A–C and Figure 8A–C). In this study, we used a serum sample pooled from 10 individual serum samples with clinically confirmed cashew sensitization and/or allergy. The SDS-PAGE and immunoblot analysis (Figure 7 and Figure 8) provided insights into the specific IgE-binding profiles of raw, roasted-, and boiled-cashew and -pistachio allergens.

As shown in Figure 7, the electrophoretic patterns of cashew protein extract from both raw and boiling-treated samples (up to 2 h boiling) were generally similar, with only a few high molecular weight bands (Ana o 1) showing potential denaturation and precipitation, as demonstrated in Figure 7A (Lanes 1–5). However, it was evident from Figure 7A that the bands corresponding to Ana o 1 and o 2 nearly disappeared in cashew kernels boiled for 4 h. Consequently, the soluble fraction of cashew boiled for 4 h was scarcely recognized by sIgE, as illustrated in the immunoblot image (Figure 7B). Interestingly, the Ana o 3 allergens demonstrated strong resistance to boiling even for 4 h in SDS-PAGE, with two district bands (Figure 7A) showing the IgE binding to only one of these bands at 10 kDa. (Figure 7B).

In addition to the SDS-PAGE and immunoblot analysis, the dot blots showed a significant reduction in IgE-binding with boiling time, evidenced by decreased dot intensities (Figure 7C). Notably, the 4 h-boiled cashew protein showed the lowest intensity. On the other hand, soaking did not induce significant changes in the IgE-binding capacity of cashew protein. Interestingly, the roasted cashew protein seemed to exhibit the highest intensity, indicating the highest IgE-binding capacity among all samples. All the water samples showed minimal to no binding (Figure 7C).

For pistachio, as apparent from the SDS-PAGE and immunoblot images, Pis v 5 bands at 36 kDa were the first to be impacted after 1 h of boiling (Figure 8A,B). Overall, the bands of treated pistachio protein decreased with increased boiling time, with almost all bands disappearing after 4 h of boiling, except for a few lower-molecular-weight bands (Figure 8A, Lane 6). Similarly, the intensity of the IgE binding decreased as the boiling time increased (Figure 8B). A significant reduction in IgE binding was observed after 2 h of boiling (Figure 8B, Lane 5), with only a faint IgE-reactive band at 36 kDa detected after 4 h of boiling (Lane 6). The IgE binding to 2S albumin bands was not detected in this study.

Similarly to the cashew proteins, the roasted pistachio protein seemed to exhibit the strongest IgE-binding for all the visible allergens. Notably, very weak bands were observed in the SDS-PAGE, but no notable bands were detected in immunoblot of the water samples, either due to low quantity or loss of IgE epitopes (Figure 8A,B). The dot blots of pistachio proteins revealed that overall, soaking did not significantly affect the IgE-binding capacity (Figure 8C). However, boiling induced loss of the IgE-binding capacity with increased boiling time, through a potential loss of epitopes due to protein modification.

These results align with previous findings obtained under harsh thermal treatments, such as autoclaving at 121 °C for 0.5 h, autoclaving at 138 °C for 15 min, and dry, dark roasting for 24 min at 149 °C for 24 min, which similarly reduced the IgE-binding capacity of cashew allergens [25,28,29]. Steam roasting and high-temperature–long-time autoclaving have also demonstrated noticeable effects in reducing the IgE-binding capacity of pistachio allergens [25,30]. The previous studies, however, only demonstrated boiling of cashew and pistachio for up to 1 h, resulting in a limited reduction in the IgE-binding capacity of these two nuts [25]. The remarked reduction in IgE-binding capacity observed after 4 h of boiling was attributed to protein fragmentation and conformational changes in allergens in both cashew and pistachio. However, it is important to note that this study is limited by the small sample size and the exclusive use of serum from cashew-allergic patients and animals, as serum from pistachio-allergic subjects was unavailable.

Drawing from the successful outcomes of the Boiled Oral Peanut Immunotherapy (BOPI) trial, which established the efficacy of boiled peanuts in reducing allergenicity and facilitating desensitization, this study investigated similar thermal-processing techniques for cashew and pistachio nuts. Our study evaluated the impact of extended boiling on these nuts to determine its potential for modifying allergenic properties and developing safer therapeutic options. The results demonstrated that boiling cashew and pistachio nuts for up to 4 h significantly reduced their allergenic potential by altering protein structures and decreasing allergen-specific IgG and IgE binding. This study shows considerable promise for oral immunotherapy (OIT), providing a viable method for gradual desensitization with allergens of reduced potency. Future studies should focus on optimizing these boiling conditions and assessing their practical applications in hypoallergenic food products to further enhance OIT outcomes.

## 4. Conclusions

In the current study, extended boiling up to 4 h modified the solubility, conformational structure, as well as dramatically reduced the polyclonal IgG- and sIgE-binding capacity of allergens in both cashew and pistachio nuts. Short soaking did not show any significant effects on the IgG- and IgE-binding capacity of the allergens. Roasting appeared to increase the IgE-binding capacity of allergens in both cashews and pistachios. Boiling the cashew and pistachio kernels effectively removed significant quantities of highly potent 2S albumins from the nuts, gradually reducing their allergenic potential with longer boiling times, resulting in progressively lower allergenicity. Pistachio proteins appeared to be more sensitive to boiling than cashew proteins. The boiling regime (i.e., boiling for 0.5, 1, 2, and 4 h) has potential for use in cashew and pistachio oral immunotherapy. Additionally, the cross-reactivity between cashew and pistachio, and their processing-induced allergen modification demonstrated in this study, suggest that boiled pistachio proteins with even lower allergenic potential could be used as a cashew substitute in cashew OIT.

## Data Availability

The original contributions presented in the study are included in the article/Supplementary Materials, further inquiries can be directed to the corresponding author.

## Supplementary Materials

The following supporting information can be downloaded at: https://www.mdpi.com/article/10.3390/foods13213440/s1, Table S1. Information on the patient serum samples.
