# Supplementary material for: Development of Cashew and Pistachio Ladders through a Food-Processing Approach
Source: Foods. 2024 Oct 28;13(21):3440. doi: 10.3390/foods13213440 (PMC11544924; doi:10.3390/foods13213440)
Supplement: Supplementary file 1 [file foods-13-03440-s001.zip › foods-3210351-supplementary.pdf]

Supplementary. Table.S1. Information on the patient serum samples.

Sample ID	Age/Sex	Allergic/ Sensitised	Diagnosed (SPT, History/OFC)	SPT (mm)	sIgE (kU/L)
BO16	14/F	Allergic to cashew	Prev reaction to Cashew	Cas 11mm Pist 9mm	-
BO27	13/M	Allergic to pistachio	Prev reaction to Pist	Cas 7mm Pist 4mm	-
BO30	8/M	Sensitised to cashew and pistachio (allergic to peanut)	SPT	Cas 15mm Pist 10mm	-
BO33	13/F	Sensitised to cashew and pistachio (allergic to peanut)	SPT	Cas 9mm Pist 5mm	-
BO39	8/F	Sensitised to cashew and pistachio (allergic to peanut)	SPT	Cas 5mm Pist 4mm	-
BO57	13/M	Sensitised to cashew and pistachio (allergic to peanut)	SPT	Cas 7mm Pist 12mm	-
BO77	11/M	Sensitised to cashew and pistachio (allergic to peanut)	SPT	Cas 7mm Pist 7mm	Cas 1.60 Pist 2.25
BO84	8/F	Allergic to cashew and pistachio	Prev reaction to Cas	Cas 7mm Pist 6mm	Cas 4.62 Pist 4.62
8A GJ	6/M	Allergic to cashew	SPT, History	Cas 11 Pist 9	-
NM	12/M	Allergic cashew and pistachio	History	Cas 8.5 Pist 3.5	-

-: not available, Cas: cashew, Pist: Pistachio
